# Identification of fibrosis-related genes and biomarkers in diabetic erectile dysfunction

**DOI:** 10.1093/sexmed/qfae090

**Published:** 2025-01-09

**Authors:** Wenjia Deng, Lingang Cui, Teng Li, Qingjun Meng, Taotao Sun, Penghui Yuan

**Affiliations:** Department of Urology, The First Affiliated Hospital of Zhengzhou University, Zhengzhou 450052, China; Department of Urology, The First Affiliated Hospital of Zhengzhou University, Zhengzhou 450052, China; Department of Urology, The First Affiliated Hospital of Zhengzhou University, Zhengzhou 450052, China; Department of Urology, The First Affiliated Hospital of Zhengzhou University, Zhengzhou 450052, China; Department of Urology, The First Affiliated Hospital of Zhengzhou University, Zhengzhou 450052, China; Department of Urology, The First Affiliated Hospital of Zhengzhou University, Zhengzhou 450052, China

**Keywords:** erectile dysfunction, diabetes, fibrosis, biomarkers, gene signature

## Abstract

**Background:**

Diabetic erectile dysfunction (DMED) has a high incidence and is poorly treated.

**Aim:**

This study investigates fibrosis’s genetic profiling and explores potential mechanisms for DMED.

**Methods:**

The DMED model was constructed in rats using streptozotocin. Erectile function was quantified using cavernous nerve electrostimulation. Fibrosis was evaluated using Masson’s staining. RNA-seq was employed to analyze differentially expressed genes and fibrosis-related genes (FRGs) were acquired. Function enrichment analyses were performed, and genetic interaction was analyzed. Hub FRGs were screened using machine learning algorithms and Cytoscape tools and validated in Gene Expression Omnibus databases. Moreover, biological roles and subpopulation distribution of hub FRGs were determined.

**Outcomes:**

Fibrosis–related genetic functions may play a vital role in DMED.

**Results:**

Based on comprehensive analysis, 45 differentially expressed FRGs were identified. These genes participate in regulating smooth muscle cell proliferation, vasoconstriction, and collagen-associated activities. Final analyses identified and validated a core gene signature comprising TIMP1, BMP7, and POSTN. They were closely associated with diabetic complications-related signaling pathways and extracellular matrix-receptor interaction.

**Clinical Translation:**

The identified fibrosis-related gene signature may serve as the novel biomarkers for treating DMED.

**Strengths and Limitations:**

The study is the first to investigate the genetic profiles behind fibrosis and DMED using comprehensive approaches. However, the validation is not adequate and more animal experiments are needed.

**Conclusion:**

The gene profiling and biological functions of FRGs in DMED were identified. These results broaden the understanding of fibrosis in DMED.

## Introduction

Erectile dysfunction (ED) is one of the most prevalent sexual problems affecting males; 13.1%–71.2% of males are affected by ED, and the prevalence will increase to 322 million by 2025 globally.^1^ Erectile dysfunction is described by the insufficient ability to achieve a satisfactory sexual life due to poor erection. It is a pathological vascular event mediated by neurogenic control and influenced by multiple metabolic problems.^2^ Diabetes mellitus has a significant impact on ED among the predisposing factors. Diabetic erectile dysfunction (DMED) shows intricate and complex pathological processes and responds poorly to the current medication for ED.^3^ Therefore, exploring the genetic basis and specific molecular mechanism underlying DMED urgently needs to be addressed for more effective treatment.

Fibrosis is a common pathological outcome caused by severe tissue injury or the dysregulation of wound-healing repair. Corporal erectile tissue fibrosis is a significant pathophysiologic component of ED resulting from the loss of smooth muscle cells and increased collagen deposition.^4^^,^^5^ It is a serious morphological change associated with DMED.^6^ Studies have shown elevated fibrosis accompanied by profibrotic factor release in DMED.^7^^,^^8^ However, its role in the morphological changes of penile tissues and their genetic mechanism is not fully understood. Recent bioinformatics studies reported that fibroblasts regulated the corpus cavernosum through mechanisms involving the Wnt and Notch signaling pathways.^9-11^ Erection dynamically alters the positional arrangement of fibroblasts.^10^ Therefore, exploring the genetic basis of fibroblasts and associated fibrosis might greatly contribute to DMED research. However, few studies have focused on fibrosis in DMED specifically.

This is the first study to explore fibrosis-associated biological profiles in DMED through genomic research. Significant fibrosis-related genes (FRGs) in the corpus cavernosum were identified. Biological roles and pathways associated with FRGs were analyzed. Key FRGs with valuable information were verified after step by step screening and validation. These results will enrich the molecular landscape and provide new potential targets for DMED.

## Methods

### Animal model and Masson’s staining

Thirty-eight-week-old male Sprague–Dawley rats were used in the study. The DMED models were constructed by injecting streptozotocin (60 mg/kg) intraperitoneally in 20 rats. The other 10 rats comprised the control group by injecting citric acid buffer. The rats with blood glucose levels of ˃ 16.7 mmol/L three and seven days later were recognized as the diabetic state. An apomorphine test was employed to observe the erection after normal feeding for 10 weeks. Ten rats with negative results were categorized as DMED.^3^ Then, an electrostimulation test was conducted to quantify the erectile level. The rats were fixed after anesthetization. Their carotid artery and corpus cavernosum were isolated and exposed by careful dissection. Later, a biological signaling sampling and processing system (Techman, BL-420 N, China) was used to measure and record the atrial blood pressure by intubation and intracavernous pressure (ICP) by stimulating the corpus cavernosum nerve using a biological electrode. Finally, the ratio of maximal ICP and mean atrial blood pressure (MAP) was calculated to evaluate the erectile function.^3^ The Experimental Animal Administration Committee of the Center for Disease Prevention and Control in Hubei, China, approved this study.

After the animal experiment, the corpus cavernosum was isolated and divided into three parts. The middle part was used for histology by fixing it with paraffin. The other parts were stored at -80°C for molecular experiments. Based on the relevant manufacturer’s protocols, the slices of the corpus cavernosum were stained with a trichrome stain kit (G1006, Servicebio, China). The stained slides were observed for the distribution of smooth muscle and collagen under an optical microscope. The penis fibrosis was evaluated and analyzed using Image-Pro Plus (6.0) software.

### RNA sequencing and data processing

After the animal experiment, the corpus cavernosum was isolated and stored at -80°C. The total RNA was extracted using the kits (Invitrogen, USA) and the quality was examined based on 0.8% agarose gel electrophoresis and spectrophotometry. Then, RNA with a 260/280 absorbance ratio between 1.8 and 2.2 was retained. Whole mRNAseq libraries were constructed using Hieff NGS® Ultima Dual-mode mRNA Library Prep Kit ® (Yeasen, 12310ES, China) as per the manufacturer’s protocols. Then, cDNA products were purified using the AMPure XP system (Beckman Coulter, Beverly, USA). Library fragments were enriched using polymerase chain reaction (PCR) and selected based on the fragment size of 350–550 bp. The library was sequenced using the Illumina NovaSeq 6000 sequencing platform (PE50) to generate raw reads after quality assessment by a bioanalyzer (Agilent, 2010, USA).

Raw sequencing data was filtered using the Cutadapt tool. After alignment with the rat genome using HISAT2,^12^ reference genome-guided transcriptome assembly and gene expression quantification were conducted by StringTie.^13^ Differentially expressed genes (DEGs) in the DMED and control groups were identified using the DEseq2 package with a threshold of *P* < .05.^14^ The results were visualized using the “pheatmap” package.

### Data acquisition and expression analysis

RNA expression profiles associated with DMED were collected in the Gene Expression Omnibus databases (https://www.ncbi.nlm.nih.gov/geo/) for validation. The GSE2457^15^ deposited Affymetrix data files of five ED rats with type 1 diabetes and corresponding control penile tissues. The GSE259299^9^ was a dataset involving the single-cell transcriptome atlas of the corpus cavernosum from six ED with type 1 diabetes and normal rats. The genetic profiles were visualized in the Male Health Atlas (MHA) database (http://www.malehealthatlas.cn/). FRGs were retrieved from the Fibrotic Disease-associated RNAome database (FDRdb, http://www.medsysbio.org/FDRdb). Then, the common genes from FDRdb and DEGs from the sequencing data were identified as significant FRGs in DMED.

### Functional enrichment analysis

The clusterProfiler^16^ was used to carry out functional enrichment analysis for the annotated significant genes. The biological functions of specific genes were explored in the Database for Annotation, Visualization, and Integrated Discovery (https://david.ncifcrf.gov/conversion.jsp), including the Gene Ontology (GO), Kyoto Encyclopedia of Genes and Genomes (KEGG) and Reactome. Then GO and KEGG enrichment terms were explored by R packages “clusterProfiler”. Terms with *P* < .05 were considered significant.

### Protein–protein interaction analysis

The significant DEGs were processed in the Search Tool for the Retrieval of Interacting Genes database (http://string-db.org). After removing isolated nodes, a protein–protein interaction (PPI) network was constructed and rearranged in Cytoscape 3.7.1 (https://cytoscape.org/). The cytoHubba and MCODE plugins in Cytoscape were applied to screen hub genes. The cytoHubba was used to mine the profiles by topological ranking algorithms, including MCC, DMNC, MNC, and Degree in the top 20 genes. The MCODE plugin was used to conduct module analysis to represent specific molecular complexes with default values.^17^

### Machine learning algorithms

Support vector machine-recursive feature elimination (SVM-RFE) is a machine-learning approach characterized by a sequence backward selection strategy and maximum interval criterion based on SVM.^18^ Gene expression was considered as the indicative feature in this algorithm. The features were ranked according to weight. After iteration, the optimal gene signature with the smallest error was selected as the final model. This method has a good learning ability in the case of small samples. In this study, SVM-RFE was applied using the R package “e1071”.^19^ Finally, the hub FRGs were obtained by the overlapping genes from SVM-RFE, cytoHubba, and MCODE.

### Biological analysis of hub genes

The MHA database was used to excavate the subpopulation distribution of hub genes in cell clusters of the corpus cavernosum. Single-cell gene set enrichment analysis (GSEA) was performed using the R package “clusterProfiler” with a gene list sorted by log2 fold-change to reveal the biological roles of hub genes.

### Quantitative real-time PCR

The total RNA from the rats’ corpus cavernosum was extracted as described above. Then, reverse transcription was conducted using the Servicebio® Reverse Transcription Synthesis Kit (G3330, Servicebio, China). Quantitative real-time PCR (qPCR) was operated using the Master Mix (G3320, Servicebio, China). Detailed information on qPCR primers is listed in Supplementary Table 1.

## Results

### Phenotype changes in animal model

**Figure 1:**
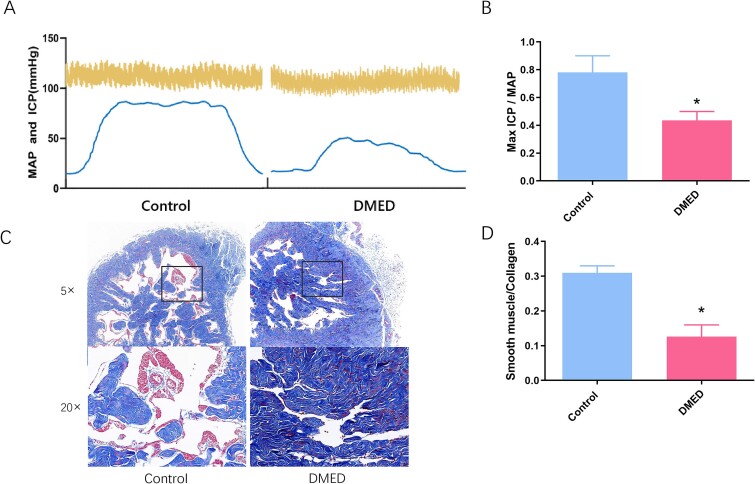
**Erectile function and fibrosis in the rats.** (A) Representative plots of ICP and MAP in the electrostimulation test. (B) Quantification of max ICP/MAP in the electrostimulation test. Representative images of the Masson's staining assay (C) and corresponding quantification (D) in the two groups. ^*^P < .05 vs. the control group. DMED = diabetic erectile dysfunction; ICP = intracavernous pressure; MAP = mean atrial blood pressure.

Compared to the control group, the ratio of max ICP and MAP decreased dramatically in the DMED group in the electrostimulation test, indicating impaired erectile function in DMED (*P* < .05, Figure 1A-B). The ratio of smooth muscle to collagen was significantly lower in the diabetic corpus cavernosum (*P* < .05, Figure 1C-D), in parallel with increased fibrosis in DMED.

### Differentially expressed DEGs analysis

After calibration and normalization, the expression distribution was found to be uniform among specimens (Supplementary Figure 1A). Differentially expressed analysis showed 1162 DEGs between the DMED and control groups, depicted as a heatmap (Supplementary Figure 1B). Among them, 498 genes were up-regulated while 664 genes were down-regulated.

### Functional enrichment analysis

**Figure 2:**
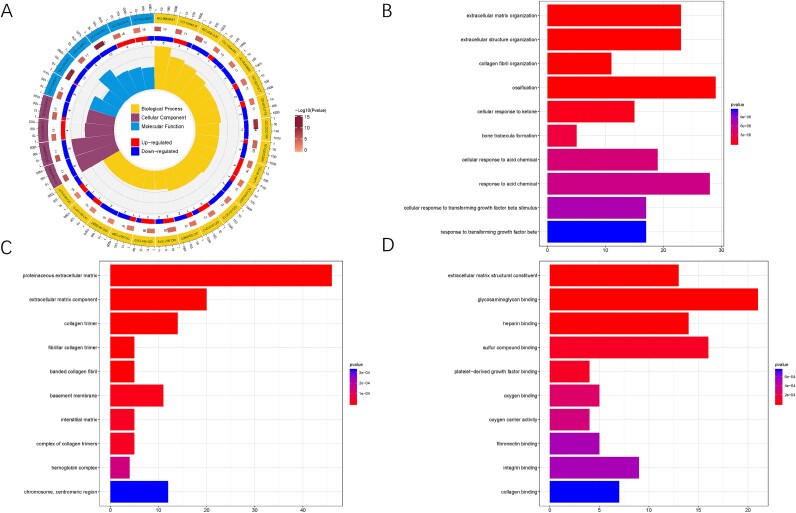
**Functional enrichment analysis of DEGs.** (A) the circle plot of GO analysis. (B) the bar plot of biological process. (C) the bar plot of cellular component. (D) the bar plot of molecular function. GO = gene ontology; DEGs = differentially expressed genes.

The biological function of DEGs was annotated using GO enrichment tools (Figure 2A). These genes participated in multiple biological processes (Figure 2B), including extracellular structure organization, collagen fibri organization, and response to TGF-β stimulus. In the cellular component, DEGs were associated with the extracellular matrix component, collagen trimer, and fibrillar collagen trimer (Figure 2C). Extracellular matrix structural constituent and glycosaminoglycan binding were enriched in the molecular function (Figure 2D). This indicates that the significant functions of DEGs were focused on the extracellular matrix, propelling further research on fibrosis in DMED.

### Identification and exploration of FRGs

**Figure 3:**
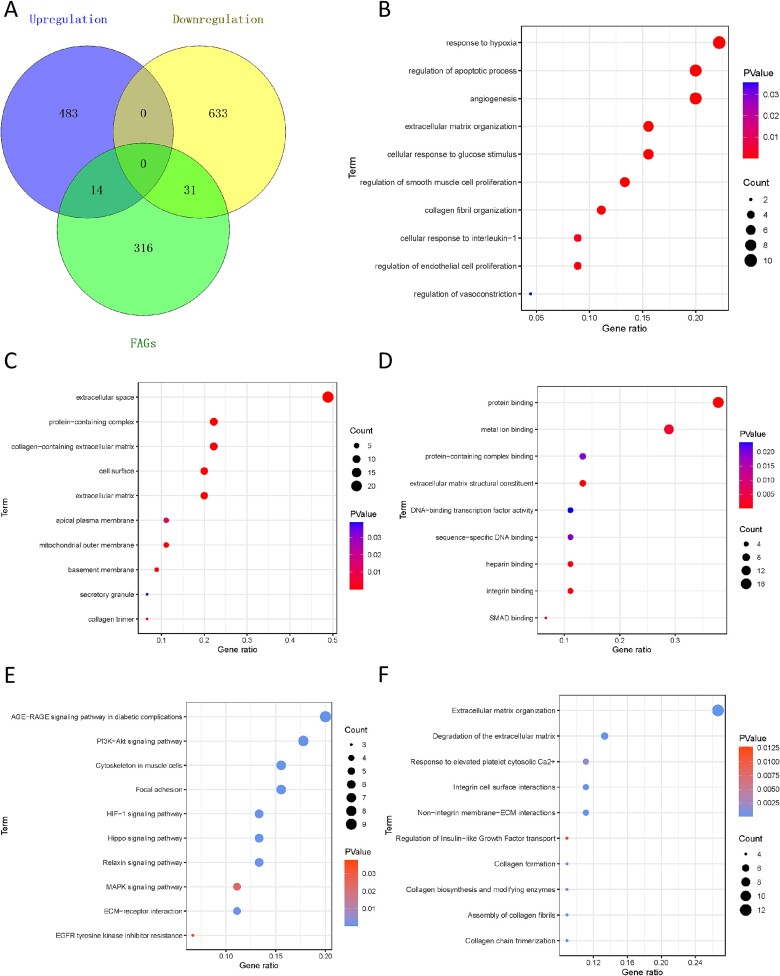
**Identification and functional enrichment analysis of** the identified **FRGs.** (A) the intersective analysis of FRGs. (B) the bubble plot of biological process in GO. (C) the bubble plot of cellular component in GO. (D) the bubble plot of molecular function in GO. (E) the bubble plot of KEGG analysis. (F) the bubble plot of Reactome analysis. FRGs = fibrosis-related genes; GO = gene ontology; KEGG = Kyoto Encyclopedia of genes and genomes.

After intersective analysis, 45 FRGs, including 14 upregulated and 31 downregulated genes, were obtained (Figure 3A). The biological process included angiogenesis, regulation of smooth muscle cell proliferation, and vasoconstriction (Figure 3B). Similarly, the extracellular space was enriched significantly in cellular components (Figure 3C). The molecular function involved protein binding and DNA-binding transcription factor activity (Figure 3D). In KEGG analysis, FRGs were involved in advanced glycation end-products (AGE)-RAGE, Relaxin, Hippo, HIF-1, and ECM-receptor signaling pathways (Figure 3E). Also, collagen-associated activities were noted in the Reactome analysis (Figure 3F).

### P‌PI network and module analysis

**Figure 4:**
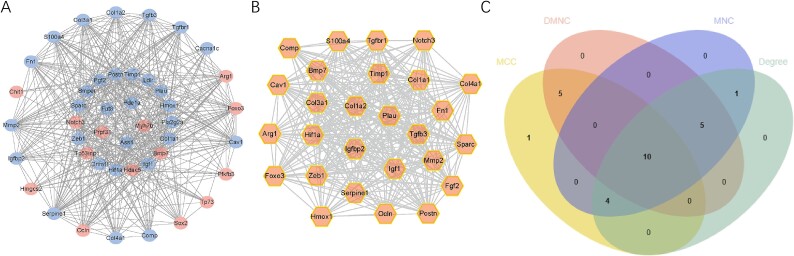
**PPI network construction and module analysis.** (A) the PPI network of significant FRGs. (B) the top gene cluster based on module analysis. (C) the intersection of genes as determined by four topological ranking algorithms in Cytohubba. PPI = protein–protein interaction; FRGs = fibrosis-related genes.

**Figure 5:**
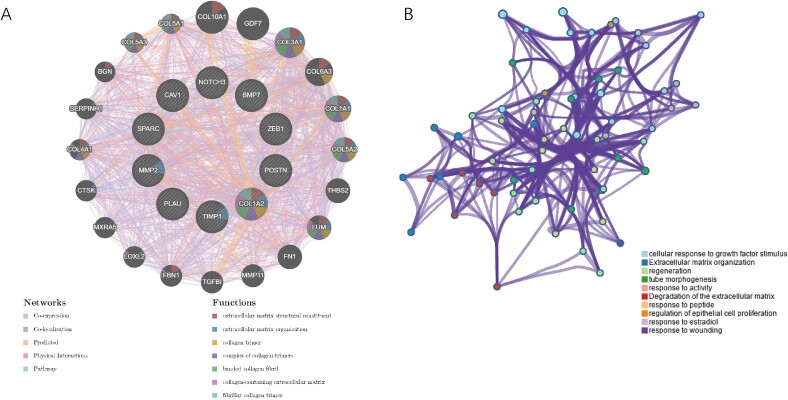
**The biological profiles of screened FRGs.** (A) the interaction patterns of FRGs in GeneMANIA. (B) the functional annotation of FRGs in Metascape. FRGs = fibrosis-related genes.

A PPI network was constructed containing 44 nodes and 449 interactions to delineate the interplays among FRGs (Figure 4A). The MCODE analysis presented 1 gene cluster with 27 genes (Figure 4B). To find the core role in this network, four topological ranking algorithms in cytoHubba were utilized to identify the most corresponding components. After comparative analysis, 10 genes exhibited great connectivity (Figure 4C). The functional annotation of these genes was performed using the GeneMANIA tool (https://genemania.org/) (Figure 5A) and Metascape (http://metascape.org) (Figure 5B). As expected, they exhibited strong association with extracellular matrix and collagen activities.

### External validation and identification of hub CPRGs

**Figure 6:**
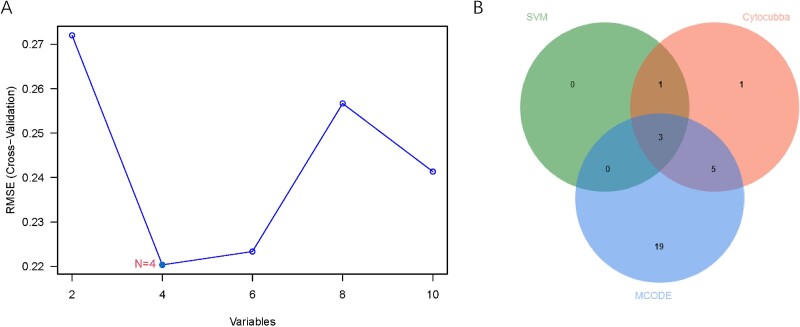
**Identification of hub FRGs.** (A) Candidate genes identified by SVM-RFE. (B) the Venn diagram showing the hub FRGs identified via SVM-RFE, cytoHubba, and MCODE. FRGs = fibrosis-related genes.

Ten genes were further validated in another DMED dataset to enhance authority. The GSE2457 and MHA analysis showed similar results (Supplementary Figure 2A-B). Herein, COL1A2, MMP2, and SPARC were predominately enriched in fibroblasts (Supplementary Figure 2C). Furthermore, using the SVM-RFE algorithm, a subset of four feature-corresponding genes among 10 genes were identified with the smallest error (Figure 6A), including POSTN, TIMP1, MMP2, and BMP7. Based on intersective analysis, the overlapping genes among SVM-RFE, cytoHubba, and MCODE were ultimately selected as hub FRGs, namely POSTN, TIMP1, and BMP7 (Figure 6B). Finally, the expression profiles of these genes were validated using qPCR. A significant difference in expression was obtained in POSTN, TIMP1, and BMP7 between the DMED group and the control group (Supplementary Figure 3).

### Exploring the functions of the hub FRGs

**Figure 7:**
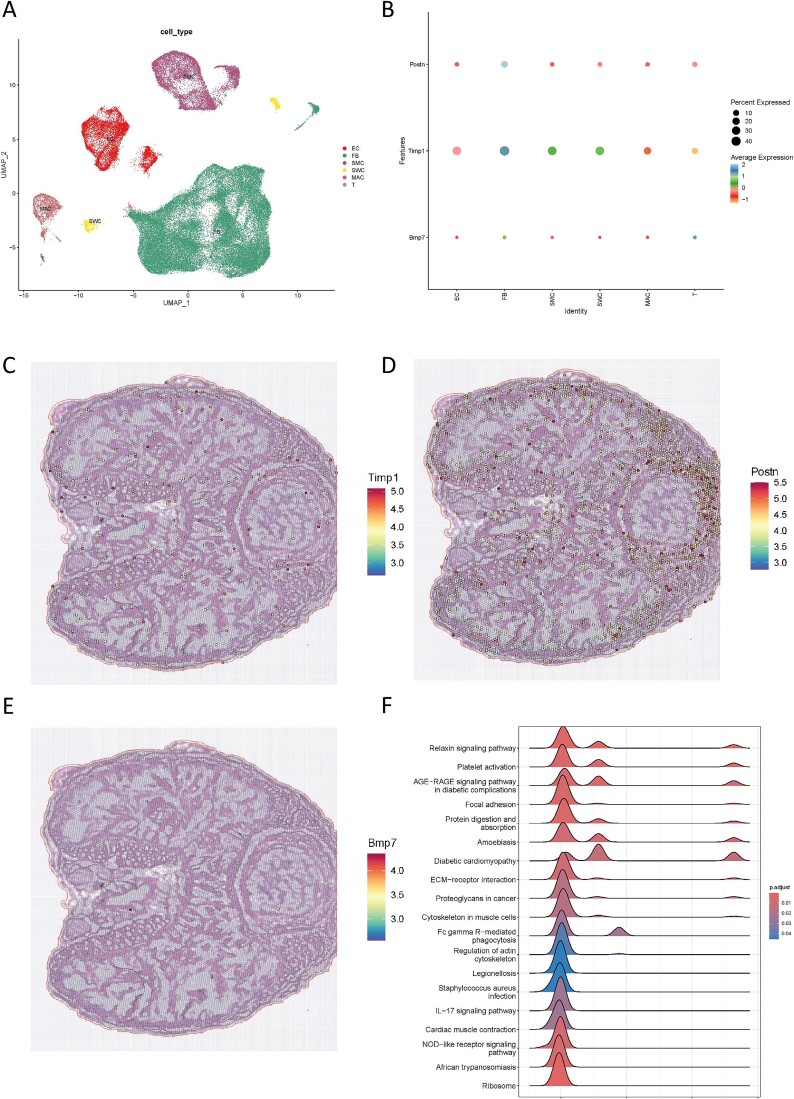
**Subpopulation distribution of hub FRGs.** (A) UMAP visualization of subpopulations clustering of the rat corpus cavernosum. (B) Expression distribution of hub FRGs grouped by cell types. The expression distribution of TIMP1 (C), POSTN (D) and BMP7 (E) in the corpus cavernosum tissues. (F) Single-cell GSEA of hub FRGs. FRGs = fibrosis-related genes; EC = endothelial cell; FB = fibroblast; MAC = macrophage; SMC = smooth muscle cell; SWC=Schwann cell; T = T cell; GSEA = single-cell gene set enrichment analysis.

The subpopulation distribution of hub FRGs in corpus cavernosum was explored in MHA. Six types of cells existed in rat corpus cavernosum for clustering analysis, comprising endothelial, fibroblast, smooth muscle, Schwann, macrophage, and T cells (Figure 7A). TIMP1 showed the highest expression (Figure 7B). As predicted, fibroblasts and extracellular matrix accounted for the predominant cellular distribution (Figure 7C-E). Based on the results of single-gene GSEA, hub FRGs were closely associated with the AGE-RAGE signaling pathway in diabetic complications and extracellular matrix-receptor interaction (Figure 7F).

## Discussion

The pathogenesis and treatment for DMED are pressing concerns in andrology. The manifestations of DMED appear earlier and more severe and affect 35%–90% of men with diabetes compared with ED arising from other organic causes.^20^ Therefore, determining the key pathogenesis of DMED is vital for effective therapies. Although penile fibrosis was noted in DMED, the genetic alternation and associated signaling pathways have been seldom studied. This study investigated the relationship between fibrosis and DMED based on multi-omics methods, and identified biological profiles related to fibrosis and the associated signaling pathways for the first time. It is significant to explore genetic alternations and promote effective treatments for DMED.

Differential expression and functional enrichment analyses using RNA sequencing showed that DEGs were involved in multiple biological processes. Extracellular collagen activities accounted for the predominant contents. These paved the way for the subsequent research in fibrosis. After the acquisition of the significant FRGs, their biological functions were explored. The smooth muscle cell’s function is the terminal process of penile erection.^3^ The loss of smooth muscle cells accompanies fibrosis. The AGE-RAGE signaling pathway is a common initial process in diabetic complications, which was studied in DMED.^21^ Subsequent biological stimuli could accelerate profibrotic factors, resulting in smooth muscle cell stiffness due to high-glucose conditions.^22^ Furthermore, extracellular matrix proteins were accumulated, mediated by AGE-modified collagen, leading to tissue fibrosis.^23^ The Hippo pathway was also explored in ED. In a model of high-fat diet-induced ED, Chen et al.^24^ reported that a high level of cytoskeletal protein SPTA1 could decrease erectile function via the Hippo signaling pathway. Similarly, the therapy of low-intensity pulsed ultrasound could improve erectile function in neurogenic ED rats associated with the regulation of YAP/TAZ-mediated mechanotransduction.^25^ Further mining is needed to determine whether it is involved in DMED. Hypoxia impairs erectile function by downregulating the expression of neuronal and endothelial nitric oxide synthases.^26^ HIF-1 is a transcriptional activator mediating cellular adaptation to hypoxia and participates in anaerobic metabolism. Wang et al.^27^ found that the HIF-1 signaling pathway was enriched significantly in DMED. Studies showed that HIF-1 suppressed the oxidative stress-induced proliferation of fibroblasts and mediated vascular remodeling in fibrosis-related diseases.^28-30^ Based on these, we hypothesized that the HIF-1 signaling pathway contributed to penile fibrosis in DMED and should be studied in the future.

Multidimensional screening and validation were used to enhance the core function of candidate genes. The SVM-RFE approach was adopted as it could get a low error rate in small-sample studies, which was suitable for the analysis of ED. Finally, TIMP1, BMP7, and POSTN were reserved and served as the hub FRGs in DMED. All of them had a tight relationship with the extracellular matrix. The results of GSEA also showed that they were closely associated with the AGE-RAGE signaling pathway in diabetic complications and extracellular matrix-receptor interaction, indicating the vital roles of fibrosis in DMED.

TIMP1 is the natural inhibitor of the matrix metalloproteinases. Its influence on ED has been studied. Gross saponins could boost erectile function by inhibiting TIMP1 expression in DMED.^31^ In a clinical study, circulating matrix metalloproteinases and endogenous inhibitors in patients with ED were measured, and higher TIMP-1 levels and lower MMP-9/TIMP-1 ratio were noted in the DMED group compared with controls.^32^ BMP7 is a secreted ligand of the TGF-β superfamily of proteins. It improves insulin sensitivity and correlates signal transduction in fat and diabetic rats.^33^^,^^34^ It could also counteract TGF-β-mediated fibrosis and contribute to renal diseases.^35^^,^^36^ The relationship between BMP7 and DMED will be studied later.

POSTN is a secreted extracellular matrix protein essential in tissue development and regeneration. The relationship between the extracellular matrix and DMED is critical, and POSTN is one of the underlying target genes that modulate the extracellular matrix.^37^ POSTN mitigates the inhibitory effects of AGEs on periodontal stem cell proliferation and oxidative injury through AGE receptors.^38^ What’s more, it could promote liver fibrogenesis by activating LOX and propel BMP-1-mediated proteolytic activation of LOX on the extracellular matrix.^39^^,^^40^ Therefore, POSTN-LOX-fibrosis could be a novel and promising target in DMED research.

This is the first study to investigate the genetic profiles behind fibrosis and DMED. There were limitations in this study. First, though qPCR was employed, the validation is not conclusive, and more molecular and animal experiments are needed. Although the associated signaling pathways were identified, the specific upstream or downstream pathways should be proved experimentally. What’s more, for consistency, the study was conducted and validated in ED rats with type 1 diabetes, restricting the generalization of predominant males with type 2 diabetes. ED research in rat models with type 2 diabetes will be focused later. These would be our research aims in larger prospective studies in the future.

The study is the first to reveal the gene profiling and biological functions of FRGs in DMED using multi-omics analyses. A core gene signature comprising TIMP1, BMP7, and POSTN was identified and validated. These results broaden the understanding of fibrosis and lay the groundwork for novel treatment in DMED.

## Supplementary Material

Suppementary_Figure_1_qfae090

Supplementary_Figure_2_qfae090

Supplementary_Figure_3_qfae090

Supplementary_Table_1_qfae090
