# Supplementary figures and images for: Identification of fibrosis-related genes and biomarkers in diabetic erectile dysfunction
Source: Sex Med. 2025 Jan 9;12(6):qfae090. doi: 10.1093/sexmed/qfae090 (PMC11710912; doi:10.1093/sexmed/qfae090)

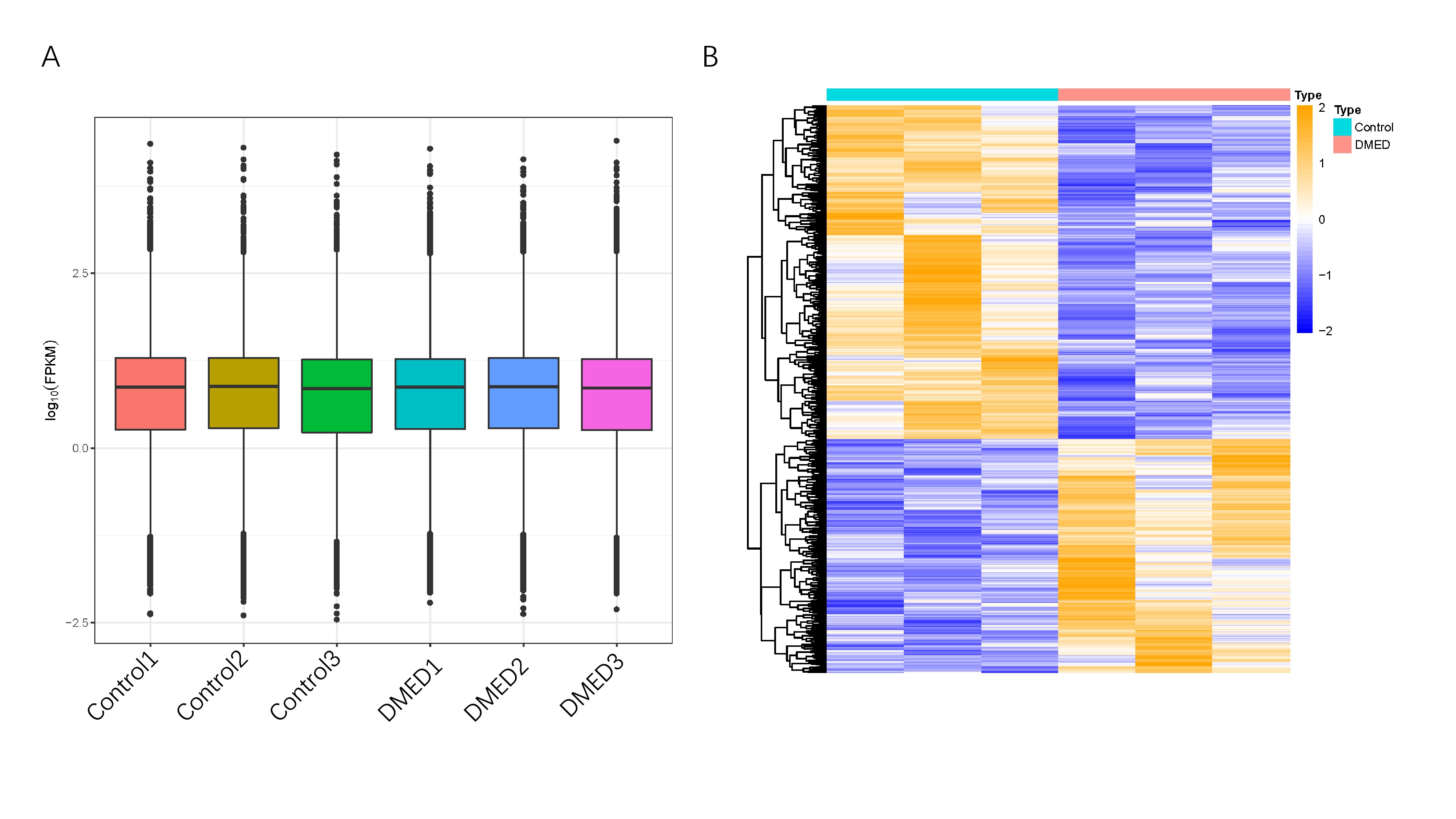

Supplement: Suppementary_Figure_1_qfae090 [file suppementary_figure_1_qfae090.jpeg]

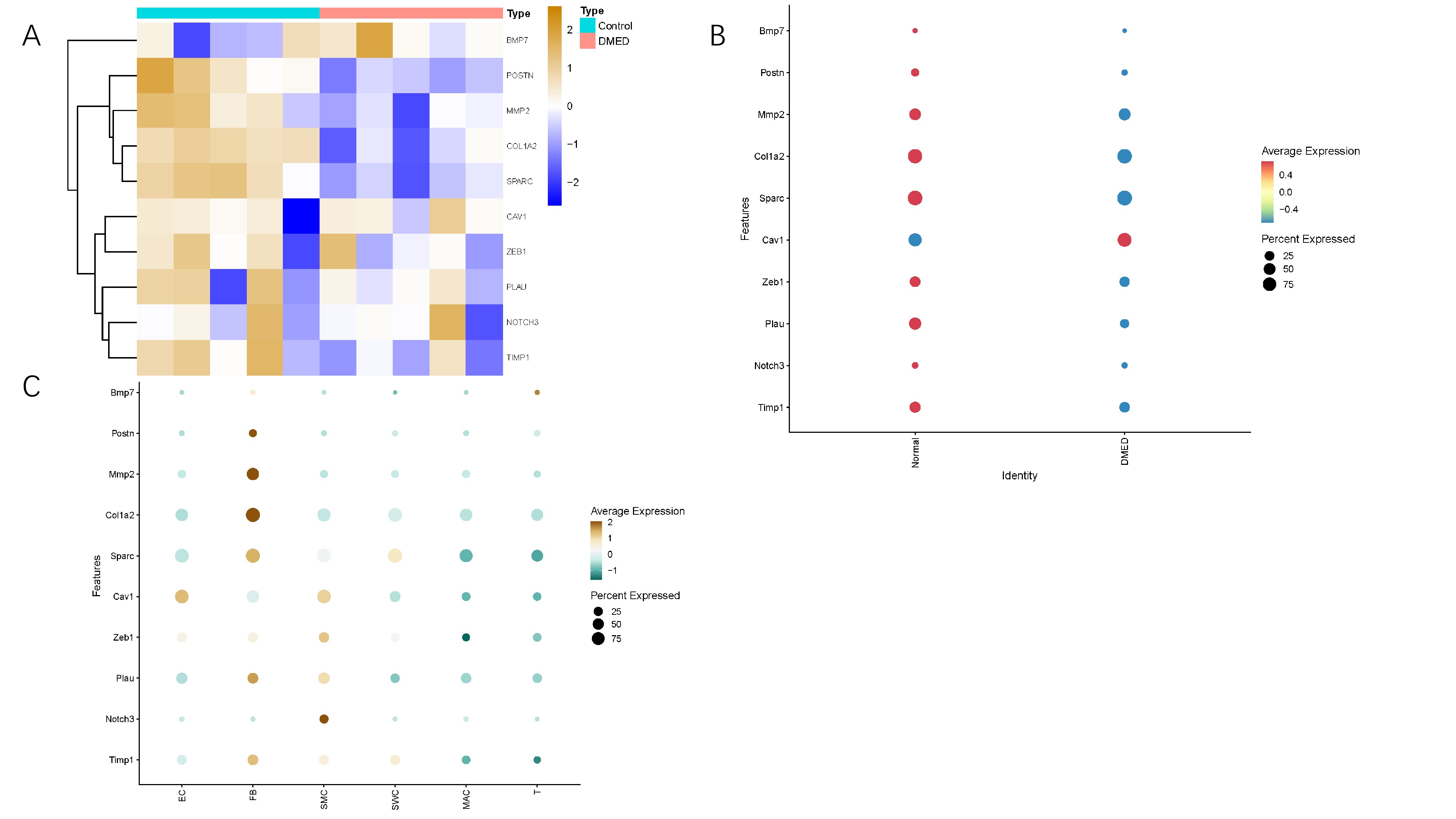

Supplement: Supplementary_Figure_2_qfae090 [file supplementary_figure_2_qfae090.jpeg]

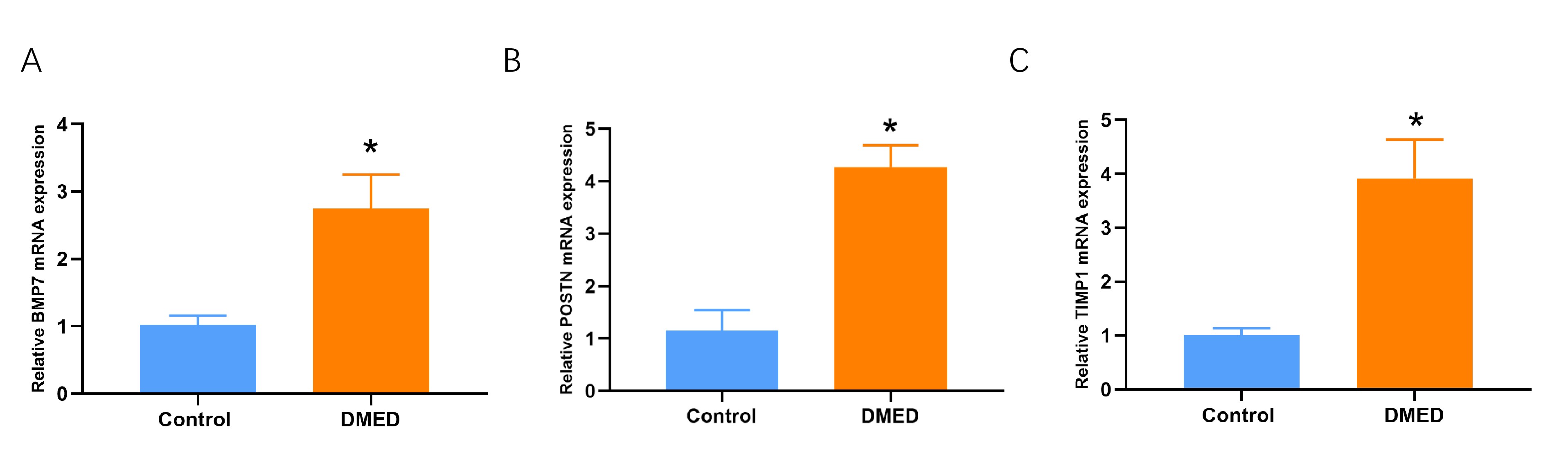

Supplement: Supplementary_Figure_3_qfae090 [file supplementary_figure_3_qfae090.jpeg]
