# Supplementary material for: Identification of fibrosis-related genes and biomarkers in diabetic erectile dysfunction
Source: Sex Med. 2025 Jan 9;12(6):qfae090. doi: 10.1093/sexmed/qfae090 (PMC11710912; doi:10.1093/sexmed/qfae090)
Supplement: Supplementary_Table_1_qfae090 [file supplementary_table_1_qfae090.docx]

**Supplementary Table 1**

The oligonucleotide sequences of primers used in quantitative real-time PCR.

| Gene | Primer type | Sequence/Target sequence |
| --- | --- | --- |
| TIMP1 | Forward | 5’-TGCAACTCGGACCTGGTTAT-3’ |
|  | Reverse | 5’-AGCGTCGAATCCTTTGAGCA-3’ |
| BMP7 | Forward | 5’-CAGCCACCAGCAACCACT-3’ |
|  | Reverse | 5’-GTCCATGCCGTCCAATCA-3’ |
| POSTN | Forward | 5’-CGCAGAGGACTAGAGAACAATGT-3’ |
|  | Reverse | 5’-AATCTGGTTCCCGTGGATTACTC-3’ |
| β-actin | Forward | 5’- GGAGCGAGATCCCTCCAAAAT-3’ |
|  | Reverse | 5’- GGCTGTTGTCATACTTCTCATGG-3’ |
